# On Some Urine Tests. II

**Published:** 1892-01-23

**Authors:** 


### ON SOME URINE TESTS.—II.

Sugar in the proportion of a tenth of a grain per ounce is shown by the ordinary Fehling's test, but care should be taken to keep the solution in a dark bottle, to boil first, then to add the urine, drop by drop, and then to boil again for a moment. If much uric acid be present, it may be necessary to precipitate it first with lead acetate, and we must also be on our guard, as Fagge remarked, for the] effects of chloroform, chloral, and salicylic acid. Probably the most delicate test after all is the fermentation one, and it has the advantage of not being affected by the presence of glycosuric acid, which reduces copper as easily as glycose does. This acid has now been found in a number of instances. Griemer employs for sugar testing a solution of safranin (1 to 1,000), which is decolourised by sugar, but not by uric acid, chloral, or kreatinin. He adds 16 drops of urine to 80 of this solution, and boils with half a drachm of the solution of caustic potash. The various improved apparatus devised of late for the fermentation tests seem inefficient, as a certain amount of the carbon dioxide given off is always re-absorbed by the urine. For those rare forms of sugar which neither ferment readily, nor reduce copper, Oliver's indigo-carmine test papers are valuable, since they are affected by the rarer forms as well as by ordinary sugar. A practical difficulty with both Fehling's solution and the fermentation test is that of getting the tests in a fresh condition. On this account Purdy's modification of Fehling is valuable. He takes 4·15 grammes of cupric sulphate, and 50 c.c. glycerine and mixes them with part of a litre of distilled water. In the remainder of the water he dissolves 20·4 grammes of caustic potash, and combining the two solutions he adds 350 c.c. of strong ammonia (sp. gr. 0·90) and filters. This gives a much more stable reagent than Fehling's tartrate of sodium and potassium, though even here we have found that a precipitate is formed after a time even in a closely stoppered bottle. The copper in one ounce of the solution should be reduced by a quarter of a grain of sugar. The picric acid test is of great delicacy and by the ingenious method of Dr. George Johnson has been converted into a quantitative one. The effect of uric acid and kreatinin both on this test and the copper sulphate may be prevented by adding beforehand to the urine 1-20 of its volume of a cold saturated solution of sodic acetate and ½ of its volume of a similar solution of mercuric chloride as was pointed out some time back in the *Dietectic Gazette.*

The tests for *acetone* can hardly be regarded as satisfactory, and, indeed, the real importance of acetone in producing the coma of diabetes is far from certain. Coma does not follow its artificial introduction into the blood, and its supposed effects have been referred to oxybutyric and other acids. However, its presence in disease may be regarded as a serious indication, and probably the test we can best rely on is its reaction with nitro-prusside of sodium and ammonium (five grains to the ounce), confirmed by the change of the purple to a greenish blue on boiling with acid. If the crystals of iodoform, which Ralfe's iodide of potash test gives, can be clearly seen, we escape the possibility, a kreatin in reaction to which the purple given by the first stage of the nitro-prusside test may be due. The ferric-chloride test is practically unreliable for the detection of acetone.

*Albumen.*—A valuable improvement in our means of discovering it has been made by the use of a solution of trichloracetic acid, which, when poured on urine, gives a cloud, not only with ordinary serum albumen, but also with modifications which are not indicated by the common re-agents. Dr. Reese found that granular epithelial and hyaline casts were present in a large proportion of the cases where the reaction was obtained, and in some he was able to verify after death the existence of changes in the kidneys. The test is, therefore, one which should not be neglected in doubtful cases. The application of heat after albumen has been precipitated by picric acid, will make the cloud thicker, and in some instances, where no precipitate is given, a cloud follows on heating. It was supposed that this was a more delicate test for albumen, but there is no evidence of casts being found in such cases; and, indeed, this reaction is probably only due to mucin-muric acid, and parapeptones are also thrown down by picric acid, but redissolved by heat. The effect of quinine must also be allowed for, and the urine should not be alkaline. With these precautions the picric acid is nearly perfect.

The old method of heating the upper layer of urine and then adding one or two drops of nitric acid has been largely superseded by the plan of treating with one-sixth of the volume of saturated solution of magnesium sulphate or common salt, to which a very little acetic acid has been added, and then applying heat. Albumen alone is thus precipitated, and as a routine test is probably only inferior to the trichloracetic acid one.

The cold nitric acid test, valuable as it is, is inconvenient, and liable to error from uric acid and copaiba, nor is subsequent heating safe, though we may thus get rid of the uric acid. The globulins are probably always associated with serum albumen, and therefore their separation, except for quantative purposes, is unnecessary. Noel Paton has proposed to precipitate them in slightly alkalised urine by saturating with sulphate of magnesium and allowing it to stand twenty-four hours. The serum albumen can then be measured by treating in the ordinary Esbach's tube, but the process is too slow a one for common use.

